# Supplementary material for: Mechanisms governing the pioneering and redistribution capabilities of the non-classical pioneer PU.1
Source: Nat Commun. 2020 Jan 21;11:402. doi: 10.1038/s41467-019-13960-2 (PMC6972792; doi:10.1038/s41467-019-13960-2)
Supplement: Supplementary file 7 — Source data [file 41467_2019_13960_MOESM7_ESM.zip › Source_Data/FigureS2/FigureS2B_MotifScanOutput/KmeansCluster11/homerResults/motif13.similar.html]

motif13

## Information for motif13

A
G
T
C
A
T
G
C
C
A
G
T
A
C
G
T
G
C
A
T
A
C
T
G
G
C
T
A
G
C
A
T
T
A
C
G
A
G
C
T
  
Reverse Opposite:  

T
C
G
A
A
T
G
C
C
G
T
A
C
G
A
T
T
G
A
C
C
G
T
A
T
G
C
A
G
T
C
A
T
A
C
G
T
C
A
G
  

|  |  |
| --- | --- |
| p-value: | 1e-34 |
| log p-value: | -8.039e+01 |
| Information Content per bp: | 1.577 |
| Number of Target Sequences with motif | 294.0 |
| Percentage of Target Sequences with motif | 9.05% |
| Number of Background Sequences with motif | 1763.5 |
| Percentage of Background Sequences with motif | 3.90% |
| Average Position of motif in Targets | 101.3 +/- 56.1bp |
| Average Position of motif in Background | 100.7 +/- 64.0bp |
| Strand Bias (log2 ratio + to - strand density) | -0.4 |
| Multiplicity (# of sites on avg that occur together) | 1.09 |
| Motif File: | file (matrix) reverse opposite |

### Similar de novo motifs found

|  |  |  |  |  |  |  |  |
| --- | --- | --- | --- | --- | --- | --- | --- |
| Rank | Match Score | Redundant Motif | P-value | log P-value | % of Targets | % of Background | Motif file |
| 1 | 0.964 | T C G A T A G C C G T A G C A T T A G C C G T A T G C A C G T A C T A G | 1e-30 | -69.289501 | 9.85% | 4.75% | motif file (matrix) |
| 2 | 0.771 | A G T C G A C T A G C T C A G T T A C G G T C A C G A T A T C G C G A T C A T G A C T G G A C T | 1e-24 | -56.913977 | 2.74% | 0.65% | motif file (matrix) |
| 3 | 0.837 | G T A C C A T G A G T C A G C T A G C T C G A T A T C G T C G A C G A T A C T G C A G T | 1e-23 | -54.003164 | 3.88% | 1.28% | motif file (matrix) |
| 4 | 0.886 | C G T A T A G C G C T A A C G T T A G C C G T A T C G A T G C A | 1e-21 | -50.363980 | 5.79% | 2.52% | motif file (matrix) |
| 5 | 0.858 | C G T A A C G T A G T C C G T A C G T A C G T A A C T G | 1e-13 | -32.153057 | 9.02% | 5.54% | motif file (matrix) |
| 6 | 0.779 | T A G C A C T G A G T C A G C T A C G T C G A T A C T G C T G A G A C T | 1e-13 | -31.343523 | 4.99% | 2.53% | motif file (matrix) |
